# Treatment intensification with radium-223 plus enzalutamide in patients with metastatic castration-resistant prostate cancer

**DOI:** 10.3389/fmed.2024.1460212

**Published:** 2024-10-24

**Authors:** Neal Shore, Joan Carles, Ray McDermott, Neeraj Agarwal, Bertrand Tombal

**Affiliations:** ^1^Carolina Urologic Research Center, Myrtle Beach, SC, United States; ^2^Vall d'Hebron Institute of Oncology (VHIO), Vall d’Hebron University Hospital, Barcelona, Spain; ^3^St Vincent's University Hospital, Cancer Trials Ireland, Dublin, Ireland; ^4^University of Utah Huntsman Cancer Institute (NCI-CCC), Salt Lake City, UT, United States; ^5^Cliniques Universitaires Saint-Luc, Brussels, Belgium

**Keywords:** radium-223, metastatic castration-resistant prostate cancer, enzalutamide, treatment intensification, combination therapy

## Abstract

Several life-prolonging therapies with diverse mechanisms of action (MoA) are available for the treatment of metastatic hormone-sensitive/castration-resistant prostate cancer, with many patients requiring multiple lines of therapy. Nevertheless, treatment optimization to further delay disease progression and improve overall survival remains an unmet need. Despite the number of agents with differing MoAs approved for advanced prostate cancer, many patients receive only one or two life-prolonging therapies. One strategy for enhancing the benefit of treatment for this aggressive disease is combining therapies with different MoAs (treatment intensification) early in the disease course, which may be more effective than administering therapies sequentially, yet still allow for subsequent sequential use of individual therapies to optimize patient outcomes. In this narrative review we discuss the rationale for combining ^223^radium dichloride (^223^Ra; an alpha-emitting radionuclide) with enzalutamide (an androgen receptor inhibitor) for treatment intensification, including their differing MoAs, their individual efficacy in this setting, and their largely non-overlapping tolerability profiles. We also summarize the preclinical and clinical data available for this combination to date, including interim safety data from the phase 3 EORTC 1333/PEACE III study which highlight the low fracture risk of ^223^Ra plus enzalutamide when administered concomitantly with bone health agents. Relevant data were sourced from clinical studies published by the authors and via searches of PubMed, clinical trial registries and congress abstracts.

## Introduction

Prostate cancer pathogenesis is significantly influenced by abnormal androgen signaling. Consequently, patients with advanced prostate cancer often receive therapies to reduce androgen ligands and downregulate the androgen-androgen receptor axis which drives tumor proliferation. While localized disease may respond to active interventions (prostatectomy, radiation), it will ultimately progress from non-metastatic hormone-sensitive to non-metastatic castration-resistant prostate cancer (nmCRPC) after androgen deprivation therapy (ADT) or metastatic hormone-sensitive prostate cancer (mHSPC) in the absence of ADT (although for some patients, prostate cancer is first diagnosed at the mHSPC stage) (1, 2). Unfortunately, most advanced prostate cancer cases eventually progress to metastatic castration-resistant prostate cancer (mCRPC), a disease stage oftentimes characterized by bone metastases (3), meaning patients are at high risk of skeletal-related events that contribute to disease-related morbidity and mortality (4, 5). Data from the Surveillance Epidemiology and End Results (SEER) program from 2013 to 2019 indicate a 5-year relative survival rate of 34% for patients whose prostate cancer has metastasized (6).

The contemporary treatment landscape for mCRPC includes multiple life-prolonging therapies with diverse mechanisms of action (MoAs). These include androgen receptor pathway inhibitors (ARPIs; abiraterone acetate [hereafter referred to as abiraterone] and enzalutamide) (7–10), chemotherapy agents (docetaxel and cabazitaxel) (11–14), poly (adenosine diphosphate-ribose) polymerase inhibitors [olaparib (15, 16), rucaparib (17), niraparib (as a fixed-dose combination with abiraterone) (18, 19), and talazoparib (20)], and targeted radionuclide therapies (^223^radium dichloride [^223^Ra] and lutetium-177 vipivotide tetraxetan [^177^Lu-PSMA-617]) (21–24). Additionally, the immunotherapies pembrolizumab (for microsatellite instability-high or mismatch repair-deficient tumors) (25) and sipuleucel-T (26) are both approved in the US only.

With the various therapeutic options available, treatment decisions can be individualized based on factors such as comorbidities, life-expectancy, disease characteristics, patient preferences, quality of life (QoL), and prior therapies (27, 28). Due to the heterogenous nature of mCRPC (29), patients may require multiple lines of therapy. However, determining optimal treatment sequences to ensure patients derive the best overall survival (OS) benefit while maintaining QoL remains challenging.

Therapies combining ADT with an ARPI and/or docetaxel have shown improved survival relative to ADT monotherapy in patients with high-risk biochemical recurrence and mHSPC (30–36). Thus, intensifying treatment by combining therapies with different MoAs (simultaneously blocking different tumor growth pathways) may benefit patients with mCRPC, potentially offering more effective disease control than sequential therapy administration. Notably, as real-world data from the US show half of patients receive only one line of therapy after mCRPC diagnosis (37, 38), using combination therapy earlier rather than later may be appropriate. As some of the life-prolonging therapies for patients with mCRPC have distinct MoAs, combining therapies may be feasible, provided the safety profile is acceptable. ^223^Ra may be particularly useful in this regard for multiple reasons. First, as ^223^Ra is an alpha particle-emitting osteotropic calcium mimetic (39), its MoA (further discussed in section 2) is distinct from that of other life-prolonging therapies approved for patients with mCRPC. Second, data from the pivotal phase 3 ALSYMPCA study indicated that ^223^Ra can be used in conjunction with best supportive care treatments (e.g., local external-beam radiation therapy, glucocorticoids, antiandrogens, ketoconazole, or estrogens) without impacting its safety profile (40). Third, an early access program suggested enhanced survival benefit when ^223^Ra was used in combination with denosumab, abiraterone, or enzalutamide relative to ^223^Ra monotherapy (41).

However, potential treatment approaches combining ^223^Ra with other approved life-prolonging therapies must be explored carefully, as a phase 3 study in patients with mCRPC and bone metastases (ERA 223) demonstrated an increased incidence of non-pathological fractures with the combination of ^223^Ra with abiraterone plus prednisone/prednisolone versus abiraterone plus prednisone/prednisolone (42). Abiraterone must be administered concurrently with corticosteroids (7, 8), which are associated with an increased risk of fractures and bone loss (43). Moreover, preclinical data highlight the bone resorptive effects of abiraterone plus prednisone which, when combined with ^223^Ra, may impair bone remodeling and suppress bone formation (44).

Although the combination of ^223^Ra with abiraterone plus prednisone/prednisolone is not suitable for patients with mCRPC due to the fracture risk [combination contraindicated in EU (7) and not recommended in US (8)], it may be feasible to combine ^223^Ra with alternative ARPIs, such as enzalutamide. Unlike abiraterone, enzalutamide does not require administration with a corticosteroid (9, 10) and inhibits androgen receptor signaling in a more targeted fashion.

## Rationale for combining ^223^Ra with enzalutamide in mCRPC

**Figure 1:**
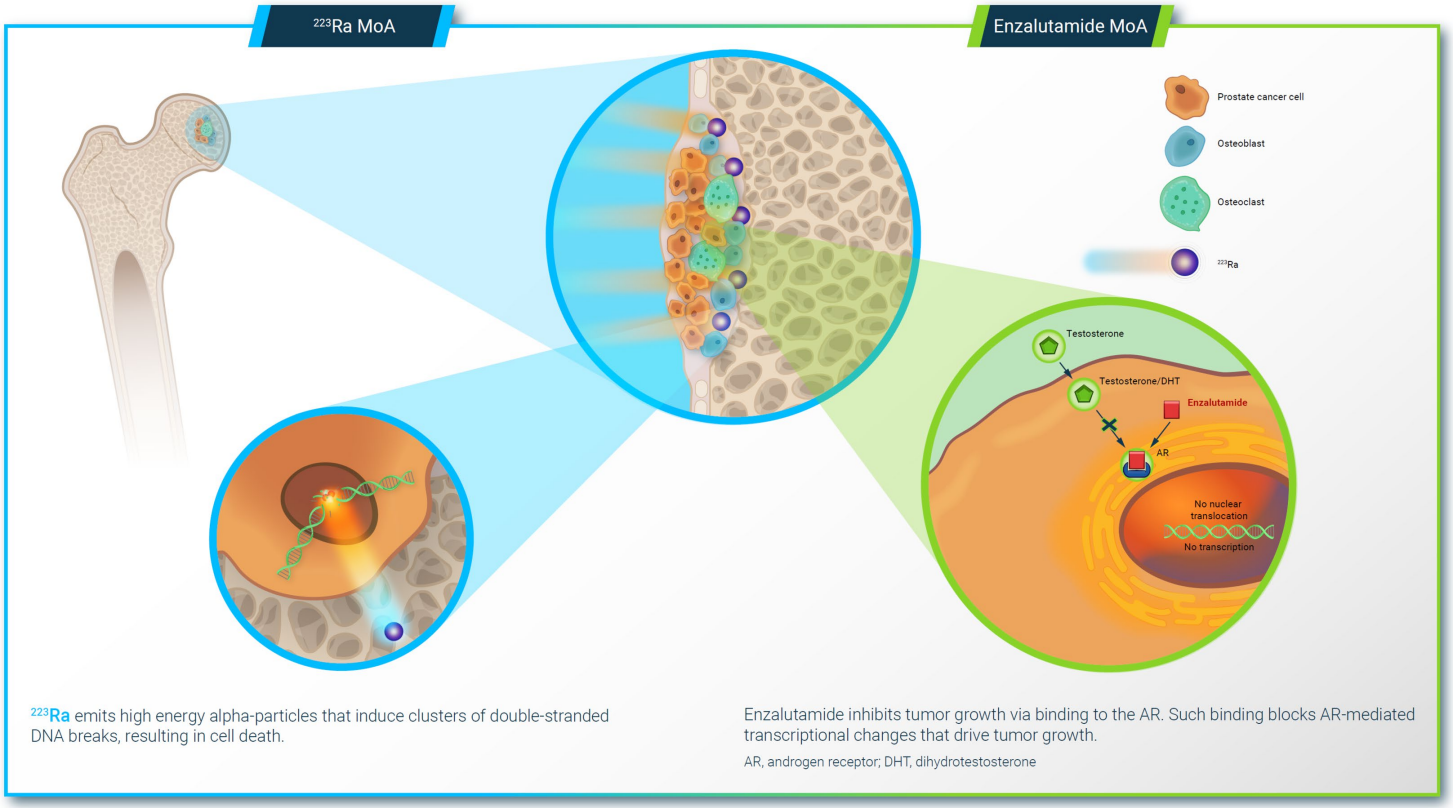
Mechanism of action (MoA) of ^223^Ra and enzalutamide.

^223^Ra and enzalutamide have distinct yet complimentary MoAs (Figure 1). ^223^Ra is a calcium mimetic that is preferentially taken up into newly formed bone within metastatic lesions, where it emits high-energy alpha particles that induce double-stranded DNA breaks within both tumor cells and nearby cells of the tumor microenvironment that contribute to metastatic growth (39). Enzalutamide directly targets the androgen receptors that drive tumor pathogenesis, competing with native androgens for androgen receptor occupancy, and blocking nuclear translocation of the androgen receptor to prevent transcription of androgen-responsive genes (45). Blocking androgen receptor function via this mechanism is a more direct method of inhibiting the androgen receptor pathway than inhibiting androgen biosynthesis (as with abiraterone) (46). Notably, androgen receptor signaling plays a role in regulating DNA repair genes (47–49), and inhibiting this pathway downregulates DNA damage repair in prostate cancer (50–52). It has been suggested that enzalutamide-mediated downregulation of DNA damage repair could sensitize cells to the double-stranded DNA breaks caused by ^223^Ra (52), leading to enhanced cancer cell death.

^223^Ra (40) and enzalutamide (53, 54) each improve survival outcomes in patients with mCRPC. In the ALSYMPCA study, ^223^Ra significantly improved median OS compared with placebo, when each was used in combination with best supportive care (14.9 vs. 11.3 months; hazard ratio [HR] 0.70; 95% confidence interval [CI] 0.58 to 0.83; *p* < 0.001) (40). Following the approval of ^223^Ra, various real-world studies have further supported the safety and effectiveness of ^223^Ra in patients with mCRPC (55–59). Regarding enzalutamide, the phase 3 AFFIRM study showed that, in patients who had previously received docetaxel, enzalutamide plus ADT significantly improved median OS versus placebo plus ADT (18.4 vs. 13.6 months; HR 0.63; 95% CI 0.53–0.75; *p* < 0.001) (53). This finding was supported by the phase 3 PREVAIL study, in which enzalutamide plus ADT provided significant improvements versus placebo plus ADT in progression-free survival (PFS) at 12 months (65% vs. 14%; HR 0.19; 95% CI 0.15–0.23; *p* < 0.001) and median OS (32.4 vs. 30.2 months; HR 0.71; 95% CI 0.60 to 0.84; *p* < 0.001) in chemotherapy-naïve patients (54).

^223^Ra and enzalutamide also have largely non-overlapping toxicity profiles, supporting the possibility of combining them without additive toxic events. The most common any-grade adverse events (AEs) with ^223^Ra in ALSYMPCA (occurring in ≥5% of patients) were bone pain, nausea and anemia. These AEs occurred at the same or higher rates with placebo and no clinically meaningful differences in the frequency of other hematologic AEs (thrombocytopenia and neutropenia) or grade 3/4 AEs were observed between ^223^Ra and placebo (40). A 3-year follow-up of ALSYMPCA indicated there were no second primary malignancies considered related to ^223^Ra and no other new safety concerns (60). In the PREVAIL (54) and AFFIRM (53) studies, the most common AEs with enzalutamide that occurred with an incidence >2% higher than with placebo included hot flashes, fatigue, gastrointestinal events and musculoskeletal events. Longer term, 5-year follow-up data from PREVAIL indicated a manageable toxicity profile for enzalutamide, although the rate of fatal treatment-emergent AEs was 1.8-fold greater with enzalutamide than placebo (6.9% vs. 3.8%) (61).

## Data for ^223^Ra in combination with enzalutamide

Data supporting the combined use of ^223^Ra and enzalutamide in patients with mCRPC can be derived from a preclinical study, several clinical trials and real-world studies. These are summarized below and detailed in Table 1.

**Table 1:** Key safety and efficacy findings for ^223^Ra plus enzalutamide in clinical trials and real-world studies.

Reference	Study design	Treatment groups (n)	Key findings	
Phase 3				
Gillessen et al. (70)	Randomized, open-label, multicenter	^223^Ra + Enz + BHA (82)	Safety	Without concomitant BHAs: two-fold increased risk of fractures with ^223^Ra + Enz vs. Enz With concomitant BHAs: fracture risk mostly eliminated with either regimen
		Enz + BHA (87)		
		^223^Ra + Enz (36)	Efficacy	Not yet available
		Enz (32)		
Phase 2				
Maughan et al. (63)	Prospective, randomized, open label, single center	^223^Ra + Enz (35) Enz (12)	Safety	Fracture incidence: 5.7% with ^223^Ra + Enz vs. 0% with Enz Several any-grade AEs were more common with ^223^Ra + Enz than with Enz (incidence difference of ≥15%), including anemia (26% vs. 7%), constipation (29% vs. 0%), diarrhea (54% vs. 7%), fatigue (46% vs. 21%), flu-like symptoms (17% vs. 0%), lymphocyte count decrease (51% vs. 29%), nausea (46% vs. 7%), neutrophil count decrease (40% vs. 0%), platelet count decrease (20% vs. 0%), and white blood cell count decrease (57% vs. 0%)
			Efficacy	Median OS: 30.8 mo (CI 17.9–NE) with ^223^Ra + Enz vs. 20.6 mo (16.8–NE) with Enz (*p* = 0.73) Median rPFS: 11.5 mo (CI 9.2–29) with ^223^Ra + Enz vs. 7.35 mo (2.8–NE) with Enz (*p* = 0.96) Median PSA-PFS2: 18.7 mo (CI 12.2–42.8) with ^223^Ra + Enz vs. 8.4 mo (CI 5.52–NE) with Enz (P = 0.033)
Shore et al. (64)	Open-label, single-arm, multicenter	^223^Ra + Enz (39)	Safety	54% of patients had TRAEs, most commonly fatigue (25.6%), nausea (17.9%) and anemia (12.8%) No serious TRAEs occurred Fractures occurred in 5.1% of patients
			Efficacy	61.5% of patients had no radiographic progression
McDermott et al. (65)	Open-label, single-arm, multicenter	^223^Ra + Enz (45)	Safety	Fractures occurred in 8.9% of patients during treatment; a further 28.9% of patients developed fractures after completing treatment, giving a cumulative incidence of 37.8% by study end No treatment-related deaths occurred Grade 3–4 TRAEs occurred in 24.4% of patients, most commonly fatigue and neutropenia (both 6.7%)
			Efficacy	Median time to PSA progression: 18.1 mo (95% CI 12.68–22.60) Median time to radiological or clinical progression: 28.0 mo (95% CI 22.54–NR) Mean time for OS: 34.8 mo (median NR)
Real-world				
Tombal et al. (66)	Prospective, multicenter, observational	Concurrent ^223^Ra + Enz + BHA (25) Layered ^223^Ra + Enz + BHA (95) Any ^223^Ra regimen + BHA (566) Concurrent ^223^Ra + Enz (21) Layered ^223^Ra + Enz (110) Any ^223^Ra regimen (899)	Safety	Fracture incidence with concurrent ^223^Ra + Enz, layered ^223^Ra + Enz, or any ^223^Ra regimen was 8% (2/25), 2% (2/95), and 3% (19/566), respectively, in patients who received concomitant BHAs and 5% (1/21), 4% (4/110), and 6% (51/899), respectively, in patients who did not receive concomitant BHAs Any-grade TRAEs occurred in 37, 28 and 35% of patients in the concurrent, layered or any ^223^Ra regimen groups, respectively; corresponding values for grade ≥ 3 TRAEs were 13, 8 and 11%
			Efficacy	Median OS: 22.2 mo (95% CI 13.7–26.8) in the concurrent group, 16.5 mo (95% CI 13.9–19.5) in the layered group and 15.6 mo (95% CI 14.6–16.5) in the any ^223^Ra regimen group
Trieu et al. (67)	Retrospective (single-center EHR data)	Concurrent ^223^Ra + Enz + BHA (33)^a^	Safety	Fractures occurred in 6.1% of patients
Shore et al. (68)	Retrospective (multicenter EHR data)	Concurrent ^223^Ra + Enz (44) Layered ^223^Ra + Enz (123) Any ^223^Ra regimen (625)	Safety	Pathological fracture incidence with concurrent ^223^Ra + Enz, layered ^223^Ra + Enz, or any ^223^Ra regimen was 9, 12, and 10%, respectively
			Efficacy	Median OS: 19.1 mo (95% CI 12.3–NR) in the concurrent group, 15.2 mo (95% CI 11.6–16.3) in the layered group and 15.2 mo (95% CI 13.2–16.3) in the any ^223^Ra regimen group

a Other treatment groups were included in this study. ^223^Ra, radium-223; AE, adverse event; BHA, bone health agent; CI, confidence interval; EHR, electronic health records; mo, months; Enz, enzalutamide; NE, not evaluable; NR, not reached; OS, overall survival; rPFS, radiographic progression-free survival; PSA-PFS2, time from start of protocol therapy to PSA progression on subsequent therapy; PSA, prostate-specific antigen; TRAE, treatment-related adverse event.

## Preclinical data

Preclinical data indicate that ^223^Ra plus enzalutamide may provide enhanced antitumor activity versus either agent alone and could potentially be used without negatively impacting bone health (52). Furthermore, enzalutamide concurrent with ^223^Ra did not alter ^223^Ra uptake in bone or the ability of ^223^Ra to reduce osteoblast number and inhibit abnormal bone formation (52).

## Clinical data

The efficacy and/or safety of ^223^Ra plus enzalutamide has been evaluated in several small (<50 patients) phase 2 clinical studies [with (62, 63) or without (64, 65) an enzalutamide comparator arm], as well as real-world studies (66–68); of these, some specify ^223^Ra and enzalutamide were administered concurrently (started within 30 days of one another) or in a layered fashion (second drug started ≥30 days after the first) (66, 68). Additionally, a randomized, multicenter, phase 3 study [EORTC 1333/PEACE III; NCT02194842 (69)] is ongoing to assess the safety and efficacy of ^223^Ra plus enzalutamide versus enzalutamide alone in patients with asymptomatic or mildly symptomatic mCRPC and bone metastases. All studies were primarily designed to assess safety (62–68), with one phase 2 trial also including change in serum levels of the bone metabolism marker N-telopeptide as a co-primary endpoint (62, 63).

## Safety

^223^Ra plus enzalutamide had an acceptable safety profile in phase 2 trials (63–65), with the largest real-world study (REASSURE) finding no new safety signals (66). In the only comparative phase 2 study, several any-grade AEs were more common with ^223^Ra plus enzalutamide than with enzalutamide alone (incidence difference of ≥15%), including anemia (26% vs. 7%), constipation (29% vs. 0%), diarrhea (54% vs. 7%), fatigue (46% vs. 21%), flu-like symptoms (17% vs. 0%), lymphocyte count decrease (51% vs. 29%), nausea (46% vs. 7%), neutrophil count decrease (40% vs. 0%), platelet count decrease (20% vs. 0%), and white blood cell count decrease (57% vs. 0%). However, these were generally grade 1–2 in severity, except for lymphocyte count decrease (grade 3 in 20% of patients) (63). In the two non-comparative phase 2 studies, the most common AEs considered to be related to ^223^Ra plus enzalutamide were fatigue (25.6 and 55.5%) and nausea (17.9 and 46.7%) (64, 65).

Given the fracture risk associated with ^223^Ra plus abiraterone plus prednisone/prednisolone (42), fractures were included as a safety outcome in several studies of ^223^Ra plus enzalutamide (Supplementary Figure S1). In an interim safety analysis of the EORTC 1333/PEACE III study, for patients who did not receive concomitant bone health agents (BHAs), a two-fold increased risk of fractures was observed in the ^223^Ra plus enzalutamide group versus the enzalutamide group at 1.5 years; however, fracture risk was mostly eliminated in corresponding groups with concomitant BHA use (70). These findings are supported by a phase 2 study in which most (96%) patients received concomitant BHAs, with the fracture incidence being 5.7% with ^223^Ra plus enzalutamide versus 0% with enzalutamide (63).

Across other phase 2 (64, 65) and real-world (66–68) studies, BHA use and fracture data were variable (Supplementary Figure S1). In a phase 2 study in patients receiving concurrent ^223^Ra plus enzalutamide, in which 38.5% received BHAs, one patient experienced worsening of a pre-existing hip fracture during treatment (deemed unrelated to therapy) and another patient experienced a hip fracture 284 days after initiating treatment; neither patient was receiving a BHA (64). When fracture incidence was assessed both during and after completing ^223^Ra plus enzalutamide combination therapy in another phase 2 study (in which 57.8% of patients were receiving BHAs at entry), the incidence was 8.9 and 37.8%, respectively (65). However, the time to first fracture event ranged from 3 to 40 months after starting treatment (65), and disease progression over time may have weakened bones in some patients, leading to fractures. Across real-world studies, fracture incidence was 2–12% in patients who received ^223^Ra plus enzalutamide in a concurrent or layered fashion (66–68) versus 3–10% with any ^223^Ra regimen (66, 68). Of note, in real-world studies, patients may not be routinely assessed for fractures unless they experience bone pain or progressive disease, so asymptomatic fractures could go undetected. This contrasts with the EORTC 1333/PEACE III trial, which includes frequent, per protocol image assessment that may identify asymptomatic fractures that would otherwise go undetected. This, along with the variation in BHA use, highlights the difficulties in comparing fracture incidence across studies.

## Efficacy

In a phase 2 study that compared ^223^Ra plus enzalutamide with enzalutamide, the combination regimen improved the pre-specified secondary endpoints of median OS (30.8 vs. 20.6 months), radiographic PFS (11.5 vs. 7.4 months) and prostate-specific antigen (PSA)-PFS (8.9 vs. 3.4 months), although the between-group differences did not reach statistical significance (63). Moreover, in a *post hoc* analysis of this trial, ^223^Ra plus enzalutamide significantly improved PSA-PFS2 (time from start of protocol therapy to PSA progression on subsequent therapy) relative to enzalutamide (18.7 vs. 8.4 months; *p* = 0.033) (63). The combination also showed promising efficacy [median OS (secondary endpoint) not reached; mean 34.8 months] (65) and improvements in QoL and pain (secondary objectives) (64) in non-comparative studies. Efficacy data for EORTC 1333/PEACE III are not yet available, although it has recently been announced that the study has met its primary endpoint (71).

In two real-world studies (66, 68) that report efficacy for ^223^Ra plus enzalutamide, median OS (from ^223^Ra initiation; secondary endpoint) was longer when the agents were administered concurrently than in a layered fashion [22.2 vs. 16.5 months (66); 19.1 vs. 15.2 months (68)]; however, these findings are limited by the small patient numbers in the concurrent groups and lack of a control group.

## Discussion

The treatment landscape for mCRPC is continuously evolving based on evidence from clinical studies, including the integration of therapies to earlier treatment lines. ^223^Ra and enzalutamide are well-established, life-prolonging therapies with strong rationale for combined use. Emerging clinical efficacy and tolerability data highlight the promise of the combination as an option for treatment intensification in patients with mCRPC. Notably, interim safety data from the EORTC 1333/PEACE III study indicate that, when taken in combination with BHAs, ^223^Ra plus enzalutamide is associated with a low risk of fractures, similar to enzalutamide alone. A UK consensus guideline strongly recommends that BHAs should be considered for all patients with mCRPC to prevent fractures (72). Similar recommendations are made by a European consensus guideline (all patients with bone metastases are advised to receive BHAs upon developing castration resistance) (73) and American Urological Association/Society of Urologic Oncology guidelines (clinicians should prescribe BHAs to all patients with mCRPC and bone metastases to prevent skeletal-related events) (28).

When choosing a therapy for patients with mCRPC, an important factor to consider is prior treatment history. With this in mind, enzalutamide plus ADT is now approved for patients with non-metastatic hormone-sensitive prostate cancer with biochemical recurrence at high risk for metastasis (9), based on findings from the EMBARK trial (36). Furthermore, there have been significant additions to the treatments recommended for mHSPC, with a shift from ADT monotherapy to ADT in combination with other agents (e.g., enzalutamide, abiraterone, apalutamide, darolutamide, docetaxel) for most patients, based on the improvements in survival seen with combination therapies (27, 28, 74, 75). Similarly, the treatment landscape for nmCRPC is also evolving, with a number of ARPIs now approved for use in this setting (9, 10, 76–80). Consequently, patients with newly diagnosed mCRPC may now have received more therapies during treatment for mHSPC or nmCRPC. For patients with mCRPC, evidence does not support the efficacy of a second ARPI following progression on a previous ARPI (27, 81, 82) and, as such, this is not recommended by the European Society of Medical Oncology or American Urological Association/Society of Urologic Oncology (27, 28). Given this, it is unclear whether ^223^Ra plus enzalutamide would be suitable for patients who have previously received an ARPI for mHSPC or nmCRPC. Notably, EORTC 1333/PEACE III excluded patients with prior enzalutamide, apalutamide or darolutamide treatment; patients with prior abiraterone use for mCRPC were also excluded, but prior abiraterone use for mHSPC was permitted [providing patients had a response or stable disease for at least 1 year in this setting (69)].

Concerns over prior ARPI use may also apply when combining an ARPI with other therapies. One such combination is enzalutamide plus ^177^Lu-PSMA-617 (another radioligand therapy), which has recently been shown to significantly improve PSA-PFS versus enzalutamide alone (13.0 vs. 7.8 months; HR 0.43; 95% CI 0.29–0.63; *p* < 0.0001) in patients with mCRPC in a phase 2 trial (ENZA-p study) (83). Similar to EORTC 1333/PEACE III, patients with prior enzalutamide, apalutamide or darolutamide were excluded from ENZA-p, although prior abiraterone use was permitted (84). Prospective studies are therefore needed to determine any impact of prior ARPI use when using combination regimens that include an ARPI.

Despite some guidelines not recommending it (27, 28), use of back-to-back ARPIs is common in some countries (38, 85). Furthermore, the proportion of patients who still receive first-line ADT monotherapy for mHSPC varies by country/region according to recent real-world studies (86–88). For instance, in a retrospective analysis of oncology patient records (2018–2020) from five countries, ADT monotherapy was the most common treatment in Western countries (53.4–58.1%), while ADT plus older anti-hormonal therapies was the most common in Eastern countries (54.6–67.2%); overall, 76.1% of patients received non-guideline-concordant therapies (87). Such findings highlight the complexity of the treatment landscape and should be considered during treatment decision-making for patients with mCRPC.

Bone metastases are common in patients with mCRPC (3), with visceral metastases typically developing later in the disease course (89). As ^223^Ra is approved for the treatment of patients with mCRPC with bone metastases and no known visceral metastases (21, 22), and enzalutamide demonstrated greater OS benefit in patients without versus with visceral metastases in the AFFIRM (53) and PREVAIL (54) trials, the combined use of ^223^Ra plus enzalutamide may be a suitable early treatment option for patients with mCRPC. ^223^Ra is also being evaluated in combination with various other agents in patients with mCRPC whose disease is confined to the bones. These include docetaxel (phase 3 DORA trial) (90), olaparib (phase 1/2 COMRADE trial) (91), nivolumab (phase 1/2 Rad2Nivo trial) (92), and ^177^Lu-PSMA (phase 1/2 AlphaBet and DUET trials) (93, 94).

To conclude, the treatment landscape of mCRPC is continuously changing. The full efficacy and safety data from EORTC 1333/PEACE III are awaited with interest, as they will help to inform clinicians as to how the combination of ^223^Ra plus enzalutamide may be used to treat patients with mCRPC.
