# Supplementary material for: Treatment intensification with radium-223 plus enzalutamide in patients with metastatic castration-resistant prostate cancer
Source: Front Med (Lausanne). 2024 Oct 24;11:1460212. doi: 10.3389/fmed.2024.1460212 (PMC11544541; doi:10.3389/fmed.2024.1460212)
Supplement: Supplementary file 1 [file Table_1.DOCX]

Supplementary Material for “Treatment intensification with radium-223 plus enzalutamide in patients with metastatic castration-resistant prostate cancer”


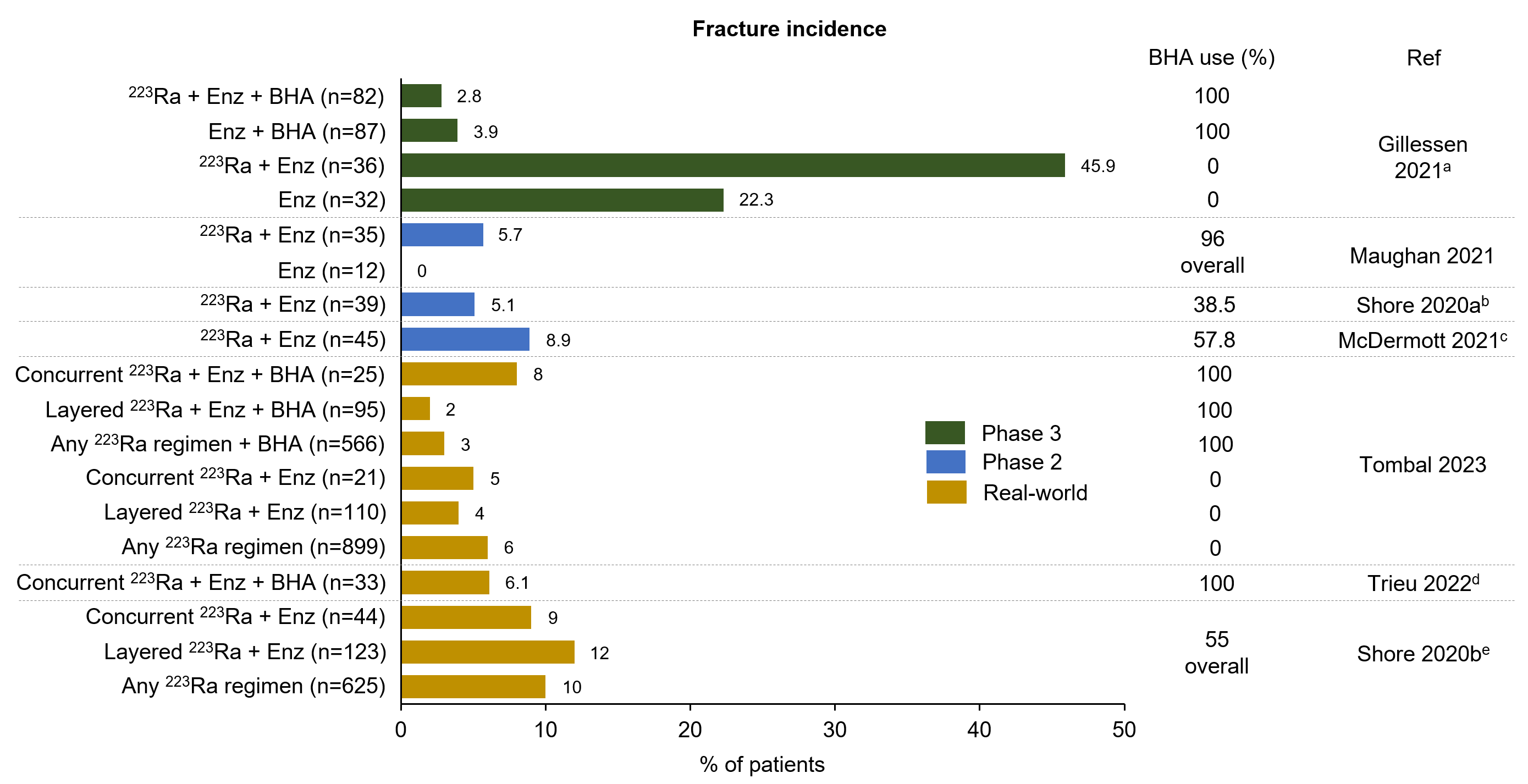


**Supplementary Figure 1. Incidence of fractures in clinical trials and real-world studies reporting the combination of ^223^Ra plus enzalutamide.** Where stated, studies evaluated any fractures (pathological or non-pathological) (Gillessen 2021, Trieu 2022, Maughan 2021), or only pathological fractures (McDermott 2021, Shore 2020b).

^a^Cumulative incidence at 1.5 years. ^b^2 patients had a fracture; neither patient was receiving a BHA. ^c^BHA use at study entry. During treatment; a further 28.9% of patients developed fractures after completing combination treatment, giving a cumulative incidence of 37.8% by study end. ^d^Other treatment groups were included in this study. ^e^55% of patients received concomitant BHAs; 67% of patients had received prior BHAs.

^223^Ra, radium; BHA, bone health agents; Enz, enalutamide.

References: Gillessen et al. 2021. J Clin Oncol; 39(15_supplement):5002. Maughan et al. 2021. Oncologist; 26(12):1006-e2129. Shore et al. 2020a. Clin Genitourin Cancer; 18(5):416-22. McDermott et al. 2021. Ther Adv Med Oncol; 13:17588359211042691. Tombal et al. 2023. Annals of Oncology; 34:S984. Trieu et al. 2022. Clin Genitourin Cancer; 20(5):399-403. Shore et al. 2020b. Prostate Cancer Prostatic Dis; 23(4):680-8.
